# Tolerance and Persistence of *Pseudomonas aeruginosa* in Biofilms Exposed to Antibiotics: Molecular Mechanisms, Antibiotic Strategies and Therapeutic Perspectives

**DOI:** 10.3389/fmicb.2020.02057

**Published:** 2020-08-27

**Authors:** Anaïs Soares, Kévin Alexandre, Manuel Etienne

**Affiliations:** ^1^GRAM 2.0, EA 2656, Normandie University, UNIROUEN, Rouen, France; ^2^Infectious Diseases Department, Rouen University Hospital, Rouen, France

**Keywords:** biofilm, *Pseudomonas aeruginosa*, antibiotic treatment, tolerance, persister cells, adaptation

## Abstract

*Pseudomonas aeruginosa* biofilm-related infections are difficult to treat with antibiotics. Along the different layers of the biofilm, the *P. aeruginosa* population is heterogeneous, exhibiting an extreme ability to adapt his metabolic activity to the local microenvironment. At the deepest layers of the biofilm is a subset of dormant cells, called persister cells. Though antimicrobial failure might be multifactorial, it is now demonstrated that these persister cells, genetically identical to a fully susceptible strain, but phenotypically divergent, are highly tolerant to antibiotics, and contribute to antimicrobial failure. By eradicating susceptible, metabolically active cells, antibiotics bring out pre-existing persister cells. The biofilm mode of growth creates microenvironment conditions that activate stringent response mechanisms, SOS response and toxin-antitoxin systems that render the bacterial population highly tolerant to antibiotics. Using diverse, not standardized, models of biofilm infection, a large panel of antibiotic regimen has been evaluated. They demonstrated that biofilm growth had an unequal impact of antibiotic activity, colistin and meropenem being the less impacted antibiotics. Different combination and sequential antimicrobial therapies were also evaluated, and could be partially efficient, but none succeeded in eradicating persister cells, so that non-antibiotic alternative strategies are currently under development. This article reviews the molecular mechanisms involved in antibiotic tolerance and persistence in *P. aeruginosa* biofilm infections. A review of the antimicrobial regimen evaluated for the treatment of *P. aeruginosa* biofilm infection is also presented. While tremendous progress has been made in the understanding of biofilm-related infections, alternative non-antibiotic strategies are now urgently needed.

## Introduction

Due to more frequent use of medical implanted devices (i.e., pacemaker, prosthesis, catheter) the burden of biofilm-related infections increased last decades (Costerton et al., 1999; Donlan, 2001; Del Pozo, 2018; Stewart and Bjarnsholt, 2020). Even when pathogens are categorized susceptible to antibiotics by routine *in vitro* testing, these infections are difficult to cure with antibiotics. Bacterial biofilms can survive antibiotics due to impaired antibiotic diffusion, antibiotic efflux, nutrient and oxygen limitation, expression of biofilm-specific genetic mechanisms, selection of resistant mutants, or survival of tolerant cells (Fauvart et al., 2011; Lebeaux et al., 2014; Harms et al., 2016; Hall and Mah, 2017; Olivares et al., 2020). While resistance is supported by *de novo* mutation or horizontal gene transfer, and is associated with an elevated antibiotic MIC, tolerance is defined by the capacity of bacteria to survive (higher minimal duration killing of 99%) despite antibiotic exposure at concentrations above MIC, without any change in the MIC (Brauner et al., 2016; Pang et al., 2019). In the stress conditions of biofilm growth, some authors consider tolerance as the predominant cause of long-term bacterial survival and antibiotic failure (Ciofu and Tolker-Nielsen, 2019).

*Pseudomonas aeruginosa* is an opportunistic pathogen frequently involved in biofilm-related infections. Though *P. aeruginosa* has largely been used as a study model to explore tolerance mechanisms and to investigate treatment strategies in biofilm, there is to date, no guidelines for the treatment of *P. aeruginosa* biofilm infections. Moreover, most of *in vitro* data on persister cells come from studies of *Escherichia coli*.

In this context, we reviewed, in *P. aeruginosa* biofilm-related models, the molecular mechanisms underlying tolerant phenotypes responsible for antibiotic failure, and the different antibiotic approaches proposed to overcome these mechanisms. Neither the non-antibiotic alternatives, nor the combinations of antibiotics and non-antibiotic compounds, to treat *P. aeruginosa* biofilms, were not voluntarily discussed in this article to focus on antibiotic strategies.

## Why a Subset of Bacterial Cells Survives in Biofilms?

### Biofilm: A Heterogeneous and Dynamic Environment

As stated by Allison et al. (2011), even when composed of a unique bacteria species, the biofilm population cannot be considered as a homogeneous entity but rather as a set of several subpopulations. Schematically, two distinct subpopulations can be described in *P. aeruginosa* biofilms: a stalk-forming subpopulation situated at the substratum, and a cap-forming subpopulation on top (Haagensen et al., 2017). But as the different layers of the biofilm progressively grow, a gradient of nutrients and oxygen leads to phenotypic and metabolic bacterial diversity (Borriello et al., 2004; Werner et al., 2004; Williamson et al., 2012). As a consequence, gene expression and phenotypic characteristics are likely to vary inside the biofilm, both within the structure and over time (Lenz et al., 2008; Soares et al., 2019b). Therefore, a continuum of quasi-independent bacterial populations exhibiting different phenotypes (i.e., susceptible, resistant and tolerant cells) may co-evolve in biofilm, and differences in the spatiotemporal organization of cell death and antibiotic tolerance development within the biofilm are observed (Haagensen et al., 2017). But, molecular mechanisms and pathways explaining how and why some subsets of biofilm cells have evolved and survive antibiotics while others will be rapidly killed by antibiotics, remain incompletely elucidated (Allison et al., 2011).

### More Tolerant Cells Than Resistant Ones: The Key Role of Persisters

**FIGURE 1:**
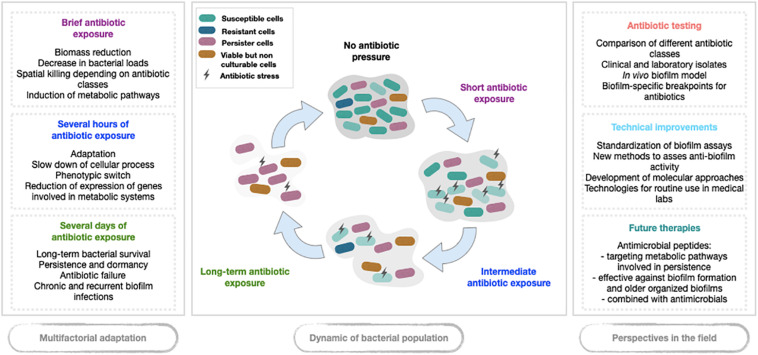
Schematic overview of phenotypic switch in biofilm under antibiotic pressure.

Acquired resistance, usually related to genetic modifications, appears not to be the main pathway of antibiotic failure in biofilm, while tolerance seems to play a major part (Ciofu et al., 2017; Hall and Mah, 2017; Peyrusson et al., 2020). Indeed, studies evaluating bacterial killing in biofilm models exposed to antibiotics showed that, at first, most susceptible cells die. The surviving cell fraction then reaches a plateau (Kamble and Pardesi, 2020). This subpopulation is not made of resistant mutants but of tolerant cells called persister cells, a subpopulation exhibiting a transient reversible phenotype allowing (i) to survive significant antibiotic exposure without MIC change and (ii) to regrow after antibiotic exposure (Lewis, 2010; Balaban et al., 2019). Such a persistent phenotype could be a consequence of biofilm organization. Stressful biofilm environment sometimes associated with antibiotic pressure might reveal a subpopulation of persister cells that was initially present. Indeed, transcriptomic analysis of bacterial biofilm under antibiotic pressure showed that bacteria on the top, exposed to oxygen and nutriment, have an intense gene expression, while bacteria on the bottom have reduced transcriptomic activity and enter a dormant state (Stewart and Franklin, 2008). By eradicating susceptible bacteria first, antibiotic could act as a revealer of the persister subpopulation that would pre-exist before antibiotic exposure. Thus, antibiotic treatment might be a determinant, hostile and unfavorable stressor which could eradicate susceptible non-persister cells, and turn the scale toward the metabolic pathways of persistence (Balaban, 2004). Higher antibiotic pressures (doses and exposure times) might also select susceptible cells in biofilm to lead them to a persister phenotype. The successive steps driving to persister survival in biofilms stressed by antibiotic exposure are presented in Figure 1.

### Pathways Toward Persistence in Biofilms

#### The Main Metabolic Pathways

Several regulatory pathways involved in *P. aeruginosa* persistence in biofilm have been identified through studies using mutants of genes involved in stringent or SOS response, and in Toxin/Anti-toxin (T/AT) systems (Murakami et al., 2005; Viducic et al., 2006; De Groote et al., 2009). Nutrient starvation in biofilm has been widely described as initiating the stringent response (Hauryliuk et al., 2015). Nguyen et al. (2011) infected mice with a wild-type *P. aeruginosa* strain or the corresponding mutant disrupted in *relA* and *spoT*, two genes encoding for two enzymes that regulate the (p)ppGpp metabolism, the key-component of stringent response. After 2 days, only 20% of mice survived, either infected by the wild-type or by the mutant strain. Interestingly, when mice were treated with ofloxacin, a 10% increase in survival was observed in mice infected by wild-type strain, while survival increased 50% in the Δ*relA*Δ*spoT* infected group. These results strongly suggest that a stringent response, regulated by RelA and SpoT, is involved in antibiotic tolerance. Biofilm environmental conditions could be a major trigger of stringent response, a mechanism contributing to the antibiotic tolerance of persister cells (Viducic et al., 2006).

The SOS response, which encompasses all the molecular mechanisms in response to DNA damages, also plays a role in bacterial persistence through different pathways for planktonic and biofilm culture conditions. In planktonic conditions, antibiotic exposure can induce the bacterial SOS response through the activation of the type I T/AT system. In planktonic cultures of *Escherichia coli* exposed to ciprofloxacin, knockout T/AT genes *tisAB/tisR* led to a decrease in persister cells level after 8 h of antibiotic exposure (Dörr et al., 2010). Dörr et al. (2009) hypothesized that SOS response might induce the expression of TisB toxin to a high level, which might cause a decrease in ATP cytosol concentrations, shutdown of the cellular metabolic and transcriptomic activity, finally leading to multidrug tolerance. It was also demonstrated in *E. coli* that two mutations in the *hipA* gene of the class II HipAB T/AT system led to a higher proportion of persister cells, particularly in stationary phase cultures (Keren et al., 2004; Schumacher et al., 2009, 2015). A very close finding was described more recently in *P. aeruginosa* by Li et al. (2016) in another class II HigBA T/AT system: an overexpression of *higB* gene increased by almost 1000 times the fraction of persister cells after exposure to sub-inhibitory ciprofloxacin concentrations in planktonic cultures. In biofilm conditions, Bernier et al. (2013) demonstrated in *E. coli* that ofloxacin persistence was directly related to the SOS response, and also to the maturity of the biofilm, but did not involve any of the SOS-induced toxin–antitoxin systems, as described above for planktonic conditions. To our knowledge, *P. aeruginosa* T/AT systems have not been explored in biofilms after antibiotic exposure at supra-MIC concentrations, and their role in antibiotic recalcitrance remains to be characterized.

Several others metabolic pathways might interact in response to antibiotic stress in biofilm. Stringent response might inhibit endogenous oxidative stress response. In the studies by Nguyen’s team (Nguyen et al., 2011; Khakimova et al., 2013), stringent response activation, in context of nutrient starvation, increased catalase activity. The production of hydroxyl radical was reduced and finally the cell killing by oxidative damages decreased. As already discussed by Hall and Mah (2017), quorum sensing might also be involved in antibiotic tolerance, as demonstrated in *P. aeruginosa* biofilms after tobramycin (Bjarnsholt et al., 2005; Brackman et al., 2011) and after meropenem treatments (Hazan et al., 2016). In these experiments, quorum-sensing deficient mutants had an increased susceptibility to antibiotics. Quorum-sensing, through a *P. aeruginosa* quorum-sensing-regulated molecule, might cause bacterial cell autolysis and DNA release. DNA subsequently would promote biofilm matrix and increase antibiotic tolerance. Nevertheless, studies on this topic are scarce and further works are required to better define the role that quorum sensing could play in biofilm tolerance to antibiotics.

#### Phenotypic Adaptation or Genetic Diversity?

Whether all pre-existing cells have the ability to evolve toward tolerant cells under stress conditions after a phenotypic switch, or a fraction of genetically determined cells can become predominant under antibiotic pressure is still debated. Cystic fibrosis airways exhibit very specific environmental conditions that favor biofilm bacterial growth (Sousa and Pereira, 2014). In this specific context, associated with very long-term *P. aeruginosa* colonization, patients usually undergo numerous antibiotic sequences that could favor genetic evolution of the bacterial biofilm population. Using a whole-genome analysis, Smith et al. (2006) demonstrated after 8 years of infection that the bacterial cells present in late *P. aeruginosa* infections of cystic fibrosis airways diverged from the cells originating the infections: virulence factors and DNA mismatch repair genes were mutated, resulting in the selection of “hypermutator” cells. In a shorter time-lapse, Klockgether et al. (2013) showed that genome sequences of *P. aeruginosa* strains from the airways of two patients with cystic fibrosis were almost identical, whereas transcriptome and metabolome were highly divergent, demonstrating the ability of genetically similar strains of *P. aeruginosa* to evolve and adapt to different hosts. Such finding was also demonstrated in a patient after a 2-week antipseudomonal treatment: a same strain evolved toward a more aerobic metabolism, via the downregulation of genes specifically involved in nitrogen metabolism (Fernández-Barat et al., 2017). Though not pathognomonic of persistence, similar phenotypic changes in the morphology of bacterial cells have been described in persister populations: high doses of aztreonam resulted in elongated filaments inside the biofilm (Rojo-Molinero et al., 2016); colistin persisters were elongated and had cell wall or membrane damages; DNA condensation was observed in amikacin persisters, and outer membrane vesicles were observed in ciprofloxacin persisters (Baek et al., 2020). These observations suggest that persister cells might develop through mechanisms depending on the antibiotic used, or on the environmental stress they go through.

Though the coexistence of genetic diversion and antibiotic tolerance through a phenotypic switch toward persistance has never been described, these two mechanisms should not be opposed and might even cooperate: the stressful conditions encountered in cystic fibrosis airways, the very long-term bacterial colonization of medical devices, the antibiotic pressure may favor emergence and sustainability of genetically divergent mutator strains, that might themselves promote transcriptomic changes and phenotypic diversity. To date, hypermutator strains have been described only in the very specific context of cystic fibrosis, whereas transcriptomic, metabolomic and phenotypic changes have been described in other biofilm conditions. The mechanisms underlying antibiotic tolerance might differ in these two kinds of biofilm-related infections and so the optimal antibiotic regimen might vary as well.

## Antibiotic Strategies to Treat Biofilm Infections

The main studies assessing *in vitro* antibiotic activity in *P. aeruginosa* biofilms are summarized in Table 1. Very diverse *in vitro* biofilm models have been implemented using different culture media, biofilm incubation time, antibiotic concentrations, leading to heterogeneous results. The current lack of standardization of the methods, parameters and interpretation of results, limits the application of the obtained data to the clinical setting, including the comparison of different treatment strategies (Lebeaux et al., 2013; Macia et al., 2014). In a clinical therapeutic perspective, we focused our review on studies that exposed mature biofilms at clinically relevant concentrations.

**TABLE 1:** Overview of antibiotic activity in *Pseudomonas aeruginosa in vitro* biofilms described in the literature.

**Biofilm model and methods used for antimicrobial susceptibility testing**	**Strains**	**Antibiotic exposure**	**Main results**	**References**
Flow-cell biofilms 4 days of biofilm growth Viable colony-counting Confocal Laser Scanning Microscopy (CLSM)	PAO1 and 2 *P. aeruginosa* clinical strains	Colistin (25 mg/L) + Ciprofloxacin (60 mg/L) Colistin + Tetracycline (200 mg/L) 24 h of treatment	Ciprofloxacin and tetracycline: killing of the subpopulation of metabolically active cells in the upper part of the biofilm Colistin: killing of cells exhibiting low metabolic activity located in the interior part of the biofilm Colistin or tetracycline and colistin: almost completely eradicated all biofilm cells (viable cells still recovered from biofilm in culture in low proportion)	Pamp et al., 2008
Flow-cell biofilms 2 days of biofilm growth Viable colony-counting CLSM	PAO1 and hypermutable derivative PAOMS	Ciprofloxacin (2 mg/L) 4 days of treatment	After an important initial reduction, increase of bacterial loads after 2 days of ciprofloxacin treatment for both strains Regeneration of the biofilm biomass after 2 days of ciprofloxacin Development of resistant mutants in biofilms: one-step resistant mutants for both strains and two-step resistant mutants for mutator strains	Macià et al., 2011
Biofilm microtitre assay 20h of biofilm growth	57 non-mucoid *P. aeruginosa* clinical isolates	Levofloxacin, Ciprofloxacin, Ceftazidime, Imipenem, Tobramycin, Colistin, Azithromycin 20 h of treatment	Ceftazidime and imipenem: the least effective antibiotics for prevention of biofilm formation and on formed biofilms.	Fernández-Olmos et al., 2012
Biofilms formed on cystic fibrosis (CF) human bronchial epithelial cells (CFBE cells) 6 h of biofilm growth Viable colony-counting (CFBE cells grown in 12-well plates) CLSM (CFBE cells injected in flow chamber)	PAO1 and 6 *P. aeruginosa* clinical isolates	Aztreonam (700 mg/L) Tobramycin (1000 mg/L) and combination of the two antibiotics 16 h of treatment	For PAO1: – Tolerance to aztreonam with reduction of the bacterial load ∼ 1 log_10_ cfu – Higher bacterial reduction with tobramycine ∼ 4 log_10_ cfu – Similar efficacy of the combination compared to tobramycin alone For clinical isolates: – ∼ 4–5 log_10_ cfu reduction with aztreonam for 4 isolates/6 – ∼ 4 log_10_ cfu reduction with tobramycin for 6 isolates/6 – Additive effect of the combination for 2 isolates/6	Yu et al., 2012
CDC biofilm reactor^®^ 24 h of biofilm growth Viable colony-counting	PAO1 and two colistin-susceptible multidrugresistant clinical isolates (both carbapenem resistant)	Colistin (1.25 et 3.50 mg/L) Doripenem (25 mg/L) and combination of the two antibiotics 72 h of treatment	Emergence of colistin resistance with colistin monotherapy Combination of colistin and doripenem: – higher initial killing for both isolates than monotherapy – no emergence of colistin resistance with combination of the two antibiotics	Lora-Tamayo et al., 2014
Flow-cell biofilms 2 days of biofilm growth CLSM	PA14	Ciprofloxacin At its MIC (160 ng/mL), 10xMIC and 100xMIC 24 h of treatment	No biofilm eradication Some cell death in the inner biofilm parts with ciprofloxacin treatment at 10xMIC and 100xMIC	Reffuveille et al., 2014
Seaweed alginate beads From 1 to 7 days of biofilm growth Viable colony-counting CLSM	PAO1	Tobramycin Peak concentrations (42.9 mg/L) 30 min of treatment	Reduction of bacterial killing as the biofilm formation period increased Bacterial killing ∼ 25% for a 1-day-old biofilm (99% for a planktonic culture) More dead cells with tobramycin but slight differences between treated and non-treated biofilm	Cao et al., 2015
Microaerobic or anaerobic environments mimicking biofilm conditions Viable colony-counting	Height *P. aeruginosa* clinical CF isolates	Fosfomycin (90 μg/mL) and Tobramycin (10 μg/mL) Alone and in combination	Synergistic activity of fosfomycin and tobramycin in susceptible isolates and those with low-level tobramycin resistance Emergence of resistant mutants prevented by the combination in aerobic and anaerobic environments	Díez-Aguilar et al., 2015
Flow-cell biofilms 2 or 4 days of biofilm growth Viable colony-counting CLSM	PAO1, PAOMS (hypermutable), PAOMA (mucoid), and PAOMSA (mucoid and hypermutable) and two hypermutable CF strains	Aztreonam, Tobramycin, and sequential exposure of the two antibiotics Peak concentrations of tobramycin (1000 mg/L), aztreonam (700 mg/L) 1/10-peak concentrations of tobramycin (100 mg/L), aztreonam (70 mg/L) 6 days of treatment	Tobramycin monotherapy: reduction in bacterial loads but observation of some alive cells in the inner part of the biofilm Aztreonam: lower reduction in bacterial loads but effects on biofilm biomass and/or structure (i.e., filamentation) Sequential exposure (tobramycin and aztreonam): enhancement of antibiofilm activity at peak concentrations Selection of resistant mutants only at the 1/10-peak concentrations for one clinical hypermutable isolate (no classical resistance mecanism higilighted)	Rojo-Molinero et al., 2016
Flow-cell biofilms 24 and 72 h of biofilm growth CLSM	PAO1	Meropenem Tobramycin Simulated human concentrations 24 h or 72 h of treatment	Meropenem: preferential killing of subpopulations within the mushroom cap of the biofilms Variable tobramycin spatial killing according to biofilm maturity More rapid killing with meropenem than with tobramycin Meropenem and tobramycin combination: rapid and efficient killing of totality of biofilm cells	Haagensen et al., 2017
CDC biofilm reactor^®^ 48 h of biofilm growth Viable colony-counting	3 colistin-susceptible and ceftazidime-resistant *P. aeruginosa* strains (multidrug- / extensively drug- resistant)	Ceftazidime (2 g/8 h), Meropenem (2 g/8 h), Ceftolozane-tazobactam (2–1 g/8 h) alone and in combination with colistin (3.5 mg/L) 54 h of treatment	Low killing with monotherapies Bacterial regrowth and emergence of resistance with colistin Combinations with colistin: prevention of emergence of colistin resistant-strains Colistin and meropenem: the most bactericidal therapy	Gómez-Junyent et al., 2019
Glass beads biofilms 24 h of biofilm growth Isothermal microcalorimetry Viable colony-counting	*P. aeruginosa* ATCC 27853 and clinical isolates	Fosfomcin + ciprofloxacin Fosfomycin + gentamicin Gentamicin + ciprofloxacin 24 h of treatment	Variable biofilm susceptibility among clinical isolates Fosfomycin: poor anti-biofilm activity Gentamicin + ciprofloxacin: synergistic activity (5/7 clinical isolates)	Wang et al., 2019
6-well culture plates 48 h of biofilm growth Viable colony-counting	PAO1 and *P. aeruginosa* clinical strains overexpressing efflux pumps	Ciprofloxacin (4 mg/L), Amikacin (40 mg/L) Single therapy, combination and sequential exposure 72 h of treatment	Bactericidal reduction after 8 h of antibiotic exposure A final mean 4 log_10_ cfu/mL plateau in all strains and for all regimens after 72 h of antibiotic exposure No biofilm eradication either with monotherapy, with combination or with sequential exposure, without regrowth of resistant mutants	Soares et al., 2019a

### Single Therapy

Eradication of bacterial biofilms requires considerably higher antibiotics concentrations compared to the killing of planktonic bacteria. In the study by Cao et al. (2015), more than 99% of the planktonic cells were killed by tobramycin, while in biofilm, the bacterial reduction was only 25%. Similarly, ciprofloxacin showed a remarkable bactericidal activity against *P. aeruginosa* in planktonic conditions (MIC = 0.25 mg/L and MBC = 1 mg/L), but exhibited a much lower activity in a microtiter biofilm model (minimum biofilm eradicating concentration [MBEC; the lowest concentration of an antibiotic that prevents visible growth in the recovery medium used to collect biofilm cells] = 512 mg/L) (Supplementary Table S1) (Wang et al., 2019). Besides, antibiotic *in vitro* activity against bacterial biofilm vary between isolates: ciprofloxacin MBEC against numerous *P. aeruginosa* cystic fibrosis clinical strains ranged from 4 to >1024 mg/L independently of their respective ciprofloxacin MIC (Wang et al., 2019). In a flow-cell biofilm model, tobramycin high dose (∼1000xMIC) demonstrated efficacy against a biofilm model of *P. aeruginosa* (Rojo-Molinero et al., 2016). Such high antibiotic concentrations can only be reached in rare clinical situations like in the sputum of cystic fibrosis patients after aerosolized drug administration (Ruddy et al., 2013). Nevertheless, for most antibiotic/isolate couples, the MBEC values may exceed the antibiotic concentrations that can be reached in a clinical practice.

As they act by inhibiting the synthesis of peptidoglycan, ß-lactam demonstrate more activity on dividing cells (i.e., in planktonic state) than on dormant cells (Ciofu and Tolker-Nielsen, 2019). In an *in vitro* biofilm device model, the biofilm growth had a strong impact on ceftazidime and imipenem with a 10 to 40 times increase in their minimal biofilm inhibitory concentrations (MBIC; the lowest concentration of an antibiotic that resulted in an OD_650_ nm difference of ≤10% of the mean of two positive growth-control well readings), whereas ciprofloxacin and tobramycin were less affected (2 to 4 times increase in their MBIC) (Fernández-Olmos et al., 2012). Among β-lactams, and for unknown reasons, only meropenem seems to have a sustained bactericidal activity against bacterial biofilm, as described in two different studies using *in vitro* pharmacodynamic models (Haagensen et al., 2017; Gómez-Junyent et al., 2019). Quinolones, aminoglycosides and colistin showed concentration-dependent or dose-dependent killing in biofilms with suitable MBIC/MIC ratios, respectively, of 1, 4, and 8 (Hengzhuang et al., 2011, 2012, 2013; Macia et al., 2014). Antibiotic exposure results in bacterial killing following a biphasic survival curve. After a rapid decrease, a small subset of cells reaches a plateau and survives to long-term antibiotic regimens. Indeed, confocal laser scanning microscopy studies showed that aminoglycosides, quinolones or colistin exposure strongly reduced the biomass of *P. aeruginosa* biofilms (Macià et al., 2011) but did not alter the biofilm structure (Rojo-Molinero et al., 2016). Thus, even if antibiotics triggered cell death in biofilm, they did not completely eradicate bacterial biofilms (Reffuveille et al., 2014), probably, as mentioned above, related to the heterogeneous metabolic activity within cells in biofilm environment and to the selection of persisters cells (Pamp et al., 2008; Haagensen et al., 2017).

### Combination of Antibiotics

Since monotherapy did not eradicate mature biofilms, combination of antibiotics appeared as a therapeutic option for the treatment of biofilm-related infections.

In numerous *in vitro* studies, colistin was used as the backbone of combination antibiofilm therapies. Indeed, colistin based combinations demonstrated efficacy against *P. aeruginosa* biofilms and seemed effective for the eradication of persister cells and to prevent the emergence of colistin resistant mutants (Pamp et al., 2008; Lora-Tamayo et al., 2014; Gómez-Junyent et al., 2019; Baek et al., 2020). For example, biofilm cells exhibiting low metabolic activity were killed by colistin whereas ciprofloxacin eradicated only the subpopulation of metabolically active biofilm cells (Pamp et al., 2008). Since, among β-lactam based combinations, meropenem showed the highest efficacy against bactrial biofilm (Haagensen et al., 2017; Gómez-Junyent et al., 2019), a colistin/meropenem combination would theoretically be a most attractive therapeutic option for biofilm-related *P. aeruginosa* infection. Nevertheless, due to the frequent kidney toxicity of colistin and the emergence of resistance clones to colistin (*mcr* gene) and/or meropenem (carbapenem-resistant strain) this combination may not be suitable for all biofilm-related infections (Biswas et al., 2012; Marchaim et al., 2019). Aminoglycosides are widely used to treat *P. aeruginosa* infections (Mensa et al., 2018). Some aminoglycosides-based combinations showed anti-biofilm activity. Combination of meropenem and tobramycin resulted in a rapid and efficient killing of biofilm cells, initiating from the top cell layer of the biofilm (Haagensen et al., 2017). Gentamicin and ciprofloxacin were synergistic against a biofilm of *P. aeruginosa* using clinical strains (Wang et al., 2019). Fosfomycin and tobramycin were also proved to be synergistic against cystic fibrosis *P. aeruginosa* isolates under conditions simulating microaerobic or anaerobic environments that are present in biofilm-mediated infections (Díez-Aguilar et al., 2015). In other experiments, a lack of additive effect of other combinations compared with single therapy was noticed in biofilm. In a static *in vitro* model of biofilm, the ciprofloxacin/amikacin combination had no benefit over monotherapy against laboratory and clinical *P. aeruginosa* strains in biofilm, regarding efficacy and persister eradication (Soares et al., 2019a). A lack of antibiotic efficacy was also noticed for the tobramycin/aztreonam combination therapy that exhibited an additive effect on biofilm disruption only for two clinical strains out of six (Yu et al., 2012).

Finally, though some antibiotic combinations could prevent the emergence of resistant mutants in biofilm, none has proven a total efficacy in overcoming tolerance mechanisms or in eradicating persister cells.

## Therapeutic and Technical Perspectives

Some authors proposed that antibiotic sequential exposure could eradicate persister cells. In planktonic cultures, Chung and Ko (2019) demonstrated that sequential treatment of colistin and amikacin, whatever the sequence, killed A*cinetobacter baumanii* persister cells. In a flow-cell model of biofilm study, Rojo-Molinero et al. (2016) used tobramycin as a substrate of the efflux pump MexXY-OprM, in order to increase the susceptibility to aztreonam. A sequential treatment of tobramycin and aztreonam was administered at very high concentrations in biofilm (reproducing the concentrations in the sputum of cystic fibrosis patients after aerosolized drug administration) and was more effective than monotherapy of tobramycin or aztreonam. But in another static model of biofilm, sequential exposure to ciprofloxacin and then amikacin or to amikacin and then ciprofloxacin (at antibiotic concentrations that allowed to achieve the PK/PD objectives associated with clinical efficacy after systemic administration) did not eradicate *P. aeruginosa* biofilm (Soares et al., 2019a). Besides, successive antibiotic exposures could promote an increasing proportion of persister cells, which could even become tolerant to additional antibiotic class (Wiuff et al., 2005; Michiels et al., 2016). Overall, these results suggest that a first antibiotic exposure may trigger the switch to a persister phenotype, rendering the bacterial population tolerant to all other antibiotic treatments.

Azithromycin showed potential inhibiting effects on *P. aeruginosa* biofilm with reducing of bacteria virulence factors, adhesion abilities and thus biofilm formation by inhibiting the cell-cell communication system, quorum sensing. Azithromycin seems to be of particular interest in case of cystic fibrosis to decrease long-term lung inflammation. The potential of this antibiotic is less described, and seems less appropriate, in case of biofilm device-related infection, diagnosed when biofilm is already mature (Nalca et al., 2006; Lutz et al., 2012; Olivares et al., 2020).

As a result, the non-antibiotic therapeutic perspectives, which target the metabolic pathways potentially involved in biofilm persistence, could be more relevant strategies (de la Fuente-Núñez et al., 2014; Pletzer et al., 2016). In the study by Reffuveille et al. (2014), an antimicrobial peptide, which acts by binding to the key component (p)ppGpp, could both inhibit biofilm formation and kill viable cells within a mature *P. aeruginosa* biofilm, in combination with ciprofloxacin. Such targeted approaches, though promising, have long been in the pipe-line and are still to be developed. Recent reviews (Koo et al., 2017; Defraine et al., 2018; Roy et al., 2018) offer comprehensive overviews of potential anti-persistence targets and strategies.

Nevertheless, there is a lack of high-quality clinical studies in the field of biofilm infections, both for cystic fibrosis patients and for tissue and device-related biofilm infections.

For further investigation of anti-biofilm activity of future components, it would be necessary to improve *in vitro* and *in vivo* biofilm models to monitor treatment efficacy. There is a real need for standardization of *in vitro* biofilm assays to reduce the variability in experimental results and to allow more direct comparisons between studies. New biofilm methods are required to assess the activity of future therapies but also to determine PK/PD parameters of existing antibiotics on young and old biofilms including combination antibiotic therapy (Figure 1).

## Concluding Remarks

Finally, though efficient therapeutics are still far from current practice in the medical treatment of biofilm-related infections, tremendous progress has been made during the past decade regarding the knowledge on molecular mechanisms responsible for antibiotic failure. All recent studies confirm that persister cells, exhibiting a high level of tolerance to antibiotics, play a key-role in the ability of biofilms to escape antibiotics. The molecular mechanisms involved in persistence are still partially described, but dormancy, stringent and SOS response, are major contributors to persistence. The mechanisms involved in bacterial persistence seem to differ according to the bacterial strain, and to the nature of the biofilm, hence the therapeutic approach might also need to be adapted to the specificities of the biofilm considered. When it comes to treating *P. aeruginosa* biofilm infection, we still lack standardized *in vitro* methods that would be representative of *in vivo* infections and that would allow a comparison of different regimens. Moreover, despite intense *in vitro* studies on biofilm tolerance, we are disappointed by the lack of therapeutic translation. To date, no antibiotic regimen has proven efficacy to overcome persistence mechanisms in *P. aeruginosa* biofilms, and non-antibiotic alternatives are highly expected.

## Author Contributions

AS reviewed the literature and wrote the manuscript. KA and ME reviewed the manuscript. All authors listed approved the work for publication.

## Conflict of Interest

The authors declare that the research was conducted in the absence of any commercial or financial relationships that could be construed as a potential conflict of interest.
