# Supplementary material for: Tolerance and Persistence of Pseudomonas aeruginosa in Biofilms Exposed to Antibiotics: Molecular Mechanisms, Antibiotic Strategies and Therapeutic Perspectives
Source: Front Microbiol. 2020 Aug 27;11:2057. doi: 10.3389/fmicb.2020.02057 (PMC7481396; doi:10.3389/fmicb.2020.02057)
Supplement: Supplementary file 1 [file Table_1.docx]

Table S1: Microbiological parameters to assess *in vitro* antimicrobial activity in planktonic and biofilm cultures

| **Parameter** | **Significance** | **Definition** |
| --- | --- | --- |
| MIC | Minimum Inhibitory Concentration | The lowest concentration of an antibiotic that inhibits the visible growth of a planktonic culture after overnight incubation |
| MBC | Minimum Bactericidal Concentration | The lowest concentration of an antibiotic producing a 99.9% CFU reduction of the initial inoculum of a planktonic culture |
| MBIC | Minimal Biofilm Inhibitory Concentration | The lowest concentration of an  antibiotic that resulted in an OD_650_ nm difference of ≤10% of the mean of two positive growth-control well readings |
| MBEC | Minimum Biofilm Eradicating Concentration | The lowest concentration of an  antibiotic that prevents visible growth in the recovery medium used to collect biofilm cells |

CFU: colony forming unit; OD: optical density
